# Validating Psychometric Questionnaires Using Experience-Sampling Data: The Case of Nightmare Distress

**DOI:** 10.3389/fnins.2018.00901

**Published:** 2018-12-05

**Authors:** Stefan Stieger, Tim Kuhlmann

**Affiliations:** ^1^Department of Psychology and Psychodynamics, Karl Landsteiner University of Health Sciences, Krems an der Donau, Austria; ^2^Department of Psychology, Research Methods, Assessment and iScience, University of Konstanz, Konstanz, Germany

**Keywords:** nightmare distress, validation, experience sampling, smartphone, psychometrics, questionnaire

## Abstract

Nightmares are a comparatively frequent phenomenon. They are often accompanied by emotional distress and gain clinical relevance when recurrent. To assess how much distress nightmares cause the individual, the Nightmare Distress Questionnaire (NDQ, Belicki, 1992) is probably the most often used measure. However, its validity is still disputed. To analyze the validity of the proposed three NDQ subscales in more detail, we conducted an experience sampling study, gathering data either in real-time or short retrospective timeframes over the course of 22 days twice per day (*N* = 92 participants). The measurements were implemented via a mobile app using participants’ own smartphones. Besides the dream quality, we assessed concepts on a daily basis that past research found to be related to dreams. These included critical life events, alcohol consumption, eating behavior, and well-being. We found that only the subscales “general nightmare distress” and “impact on sleep” showed convergent as well as divergent validity. The validity of the subscale “impact on daily reality perception” is unclear. If at all, this subscale is rather indirectly associated with nightmare distress. Furthermore, all of the NDQ items did not differentiate between a bad dream and a nightmare, which suggests that the NDQ might rather be a measure of negative dreams in general and not nightmares in particular. Based on the present experience sampling design, we propose to advance the validation process by further possibilities, such as an item-level, person-level, and multi-level approach. This approach seems to be especially fruitful for concepts which are not very salient (e.g., laughter), can hardly be remembered retrospectively (e.g., dream content), or are potentially threatened by recall biases (e.g., alcohol consumption).

## Introduction

Nightmares are frightening dysphoric dream sequences accompanied by feelings of fear, usually leading to the awakening of the dreamer. Interestingly, nightmares are a comparatively frequent phenomenon (Wood and Bootzin, 1990) being more prevalent in children (compared to the elderly; Salvio et al., 1992) and women (compared to men; Cuddy and Belicki, 1992; Levin, 1994). Epidemiological studies found prevalence rates of 2–6% of the population having even *recurrent* nightmares (more than once a week; Belicki and Belicki, 1982; Janson et al., 1995; Ohayon et al., 1997; Schredl, 2010).

Although nightmares are a phenomenon well known to the general public, they are often studied in connection to psycho-pathological symptoms such as sleep disturbances, PTSD, anxiety, or neuroticism (e.g., Levin, 1994; for a review, see Nielsen and Levin, 2007) to name just a few. Neurophysiological studies have found that in people with a nightmare disorder, the activation in parts of the anterior cingulate cortex and parietal lobule were increased and in the frontal and occipital gyri decreased (Shen et al., 2016). It was also shown that people with frequent idiopathic nightmares had differences in the density of slow and fast spindles compared to a control group (Picard-Deland et al., 2018). These studies using fMRI and EEG suggest that there are neurophysiological differences between people having frequent nightmares and those that do not.

Nightmares are often characterized by considerable emotional distress possibly having a dream function by regulating emotions (Blagrove et al., 2004). In general, there are several models of nightmare production (e.g., psychoanalytic models; personality and evolutionary models; neurobiological models) of which the neurocognitive model – as one of the latest suggestions – seems to integrate most of the principles of past models (Nielsen and Levin, 2007). This model assumes that nightmares are the result of a dysfunction in a network of affective processes. These processes serve as an adaptive function to extinct fear memory. If this fear extinction process is disturbed, nightmares emerge (Nielsen and Levin, 2007).

Besides these models of nightmare genesis, a large corpus of studies focuses on the *consequences* of having nightmares mostly manifested in (psychological) distress. This so-called nightmare distress has been conceptualized differently in the past (e.g., nightmare intensity, nightmare effects, nightmare related symptoms; for a review, see Böckermann et al., 2014). One conceptualization that has been frequently studied is the subjective appraisal of how much distress nightmares cause in the sufferers. To assess these subjective feelings, the Nightmare Distress Questionnaire (NDQ, Belicki, 1992) is probably the most often used questionnaire. It consists of thirteen questions with Likert-type answering options purportedly assessing trait-like distress caused by nightmares.

Although the NDQ has good reliability (Cronbach α) and is frequently used in research, its factor structure and validity is still debated. For example, Martínez et al. (2005) found three subscales in a sample of 162 university students. However, their study only included 12 participants, who reported having nightmares on a weekly basis. A more recent, well-powered study by Böckermann et al. (2014) also analyzed the factor structure of the NDQ by recruiting 213 individuals having one or more nightmares in a typical week. Again, a 3-factor solution was found in a principal component analysis (PCA) with the factors *general nightmare distress, impact on sleep*, and *impact on daily reality perception*. Although the authors found convergent as well as divergent validity between the three subscales with other nightmare related concepts (e.g., nightmare frequency, sleep quality, fear, depression) they questioned the reliability and validity of the subscale *impact on daily reality perception*. Because of these shortcomings the authors call into question if this subscale is an integral part of nightmare distress at all (Böckermann et al., 2014).

Although validation studies are important steps in developing new measures or verifying established ones, most of them follow a cross-temporal view, i.e., data are assessed at one particular point in time. For some concepts, this procedure is sufficient. But for concepts that include state aspects, a longitudinal view is necessary (e.g., Reis et al., 2016). In the present case of nightmare distress, related concepts (e.g., sleep quality, dream quality) are mostly assessed retrospectively, i.e., participants should remember how many nightmares (or other kind of dreams such as a nice dream) they had in a particular period (usually a couple of weeks). Meanwhile it is well accepted that these retrospective judgments are often biased (e.g., Schwarz and Sudman, 2012; Monk et al., 2015; Pryss et al., 2018). This especially pertains for events, which are not very salient, i.e., are not strong enough to make it into conscious awareness. Although this may not apply for nightmares because of their disturbing nature accompanied with awakening, sweating, and/or being out of breath, it might apply for other types of dreams, e.g., nice dream, neutral dream. Even for nightmares, retrospective judgments might not be accurate due to long periods that have to be judged. One possible solution are longitudinal designs. The experience sampling method (ESM) with up to several measurements per day offers the opportunity to increase the accuracy within a longitudinal framework (Mehl and Conner, 2012).

Experience sampling offers the possibility to capture participants’ everyday life behavior and has the advantage that collected data is more accurate than retrospective self-report data (Conner et al., 2009; Kurtz and Lyubomirsky, 2011; for an example about dream frequency, see Blagrove et al., 2004). Research on nightmare distress already has applied experience sampling designs (e.g., Wood and Bootzin, 1990; Köthe and Pietrowsky, 2001; Blagrove et al., 2004; Lancee and Schrijnemaekers, 2013). However, their usage is still quite rare, despite the potential to advance the field substantially (for a similar recommendation, see Nielsen and Levin, 2007).

Furthermore, using ESM offers ways for the development of measures by advancing the validation and development process by another level – the longitudinal one (for a similar argumentation, see Gillath et al., 2009). Usually, scale development and item selection is done by factor loadings, item difficulty, or stability over time when it comes to traits (usually one or two retests). With ESM designs, we have many more measurement occasions, which offers the possibility to additionally judge the deviations of measurements over time (e.g., Reis et al., 2016). For example, for a measure of state anxiety, a validation step could be to select those items which show large variation, i.e., are capable to assess a large variety of anxiety levels in everyday life. This follows the state logic of changing anxiety levels, validated in a within-person design capturing momentary changes. ESM studies are particularly powerful to investigate these changes. Furthermore, ESM designs offer the possibility to assess events (e.g., nightmares) alongside the items longitudinally to examine contextual associations (Shiffman et al., 2008). These can be used for the judgment of discriminatory power of items regarding participants’ behavior, i.e., predictive validity.

In the present study, we applied such an approach by adding an ESM based level to the cross-sectional assessment via the NDQ. We utilized the longitudinal data to examine the validity of the NDQ. First, we assessed dream quality longitudinally and analyzed whether the items and subscales of the NDQ were able to differentiate between dream qualities. Second, we aggregated longitudinally assessed events and psychological measures to create indicators that are less influenced by recall bias than retrospective judgments. We used these aggregated measures to analyze convergent and discriminant validity of the NDQ. We included variables, which did show some connection to nightmares or bad dreams in the past. For example, it has been shown that nightmares are associated with lower well-being (e.g., Levin and Fireman, 2002), occurrence of life events (e.g., Dunn and Barrett, 1988), alcohol consumption (e.g., Munezawa et al., 2011), and food intake (e.g., Nielsen and Powell, 2015). Third, we analyzed whether the NDQ had predictive validity for dream quality, taking the multilevel structure of the data into account.

## Materials and Methods

### Participants

The sample constitutes a convenience sample from a community in Germany. Research assistants recruited participants by word-of-mouth through friends, relatives, and friends-of-friends resulting in a sample size of *N* = 108. Eight participants only filled in one questionnaire (out of 44 possible ones) during the longitudinal phase and another eight participants failed to fill in the cross-sectional questionnaire. The remaining participants (*N* = 92) were mostly students (93%) with an average age of 22.9 years (*SD* = 6.9, range 17–67). Female participants comprised 71% of the sample (one participant did not disclose his/her sex).

All participants gave written informed consent prior to their participation in accordance with the Declaration of Helsinki, and guidelines of the Department of Psychology, University of Konstanz. Approval by an ethics committee was not necessary because the study did not affect the physical or psychological integrity, the right for privacy, or other personal rights or interests. Data collection was anonymous and no harmful procedures were used. Furthermore, participants were informed that they could withdraw at any time during the study without negative consequences.

### Measures

#### Daily Questionnaire

Participants had to fill in the daily questionnaire two times a day, once in the morning and once in the evening for 22 days. Most variables of interest for the current study were only assessed in the morning. These were critical life events, alcohol consumption, eating behavior, and dream quality. They were reported for the day and night prior to the assessment. The exact wording of the items was: (1) Did you have a critical life event yesterday (yes/no)? accompanied by a short definition what we meant by life event (“A situation or event, which you experienced as disturbing, traumatic, or stressful and which bothered you beyond that situation/event, for example, separation from partner, accident, job conflicts, and so forth.”); (2) Did you drink alcohol yesterday? (yes/no); (3) Did you have a feeling of fullness prior to going to bed? (yes/no); (4) How was your dream last night? (nice dream, neutral dream, bad dream without awakening, bad dream with awakening, I cannot remember; see Table 1). Well-being was assessed as a state measure at both times of the day (Diener et al., 1999). Participants had to answer the question “How is your current well-being?” [visual analogue scale from 0 = *very bad* to 100 = *very good*]. There were further questions asked, which are not part of this study (e.g., attractiveness, loneliness).

**Table 1:** Descriptives of variables under investigation.

Dream quality	Dream frequency (%)
Nice dream	243 (14.4)
Neutral dream	394 (23.4)
Bad dream without awakening	176 (10.4)
Nightmare	103 (6.1)
Don’t know	769 (45.6)
Sum	1685 (100%)
Number of days with a critical life events	*N* of participants (%)
0	35 (38.9)
1	20 (22.2)
2	13 (14.4)
3	9 (10.0)
4	5 (5.6)
>4 (max = 9)	8 (8.8)
Sum	90 (100.0)
Number of days where alcohol was consumed	*N* of participants (%)
0	6 (6.7)
1	8 (8.9)
2	5 (5.6)
3	5 (5.6)
4	9 (10.0)
5	12 (13.3)
6	8 (8.9)
7	7 (7.8)
>7 (max = 17)	30 (33.5)
Sum	90 (100.0)
Number of days with food intake before sleep	*N* of participants (%)
0	17 (18.9)
1	16 (17.8)
2	7 (7.8)
3	12 (13.3)
4	8 (8.9)
5	4 (4.4)
6	7 (7.8)
7	6 (6.7)
> 7 (max = 19)	13 (14.3)
Sum	90 (100.0)

#### Internet-Based Cross-Sectional Questionnaire After the ESM Part

In the final questionnaire, we assessed sociodemographics (age, sex, occupation), nightmare distress (NDQ) as well as further concepts which are not part of this study (e.g., Extraversion, subclinical Narcissism, Satisfaction with Life).

The Nightmare Distress Questionnaire (NDQ; Belicki, 1992) is a 13-item measure using 5-point Likert-type scales as the response format (10 items with 1 = never to 5 = always; 2 items with 1 = not at all to 5 = a great deal; 1 item with 1 = not at all interested to 5 = extremely interested). The NDQ has been proposed to measure three facets of nightmare distress (Böckermann et al., 2014). These are labeled *general nightmare distress* (NDQ General), *impact on sleep* (NDQ Sleep), and *impact on daily reality perception* (NDQ Daily Reality). We did not instruct participants to consider a specific time frame (e.g., in the last year) for their responses.

### E-Diary Procedure

The design of the study followed an experience sampling methodology (ESM; real-time and multiple time point measurements) implementing smartphones. A smartphone app was designed for this project and made freely available through the Google Play Store. Participants could directly download the app anonymously. A back-end server software realized communication with the app as well as the storage of data. When the app was opened for the first time, participants had to provide informed consent and were asked basic demographics once (age, sex, nationality). After this initial stage, the main screen appeared showing the items depending on the time of the day. Before midday the morning items were presented, after midday the evening items. Participants were reminded via text messages or WhatsApp messages to do their ratings. They filled in the items while being in their natural surroundings. The reminders were sent out twice per day for a duration of 22 days. The first daily reminder was sent out during the morning time frame between 8 a.m. and 10 a.m. and the second daily reminder during the evening time frame between 6 p.m. and 9 p.m. The reminders followed a time-contingent sampling approach, meaning they were sent at random times within time frames. The compliance rate was on average 90.3%, i.e., only about 10% of reminders were missed. Missingness for each measurement occasion was very low ranging from 1.7 to 2.4%. Missingness did not increase or decrease over time as indicated by the correlation between measurement point and percentage of missingness: Spearman *r* = -0.09, *p* = 0.56. After the ESM part of the study, the Internet-based cross-sectional questionnaire was administered. Participation was remunerated by optional entry to a raffle (two gift vouchers for 20€ each) or by course credit (for students). The entire study was run in German.

### Statistical Analyses

Our operational definition of a nightmare was a bad dream with awakening (in contrast to a bad dream without awakening). For the item-level analyses, we calculated a generalized linear mixed-effects model (GLMM; Bates et al., 2015). Occasions (level 1) were nested within persons (level 2) and the outcome was the dream quality. Dream quality was transformed into three dummy-coded variables (nice, bad, nightmare). The categories *neutral* and *don’t know* did not show substantial differences in nightmare distress and were therefore combined as the reference category. Three logistic GLMMs were calculated, testing the predictive value of the NDQ items in distinguishing the three dream qualities from the neutral reference category. The NDQ items were entered as level 2 predictors into the model and grand-mean centered (cgm; Enders and Tofighi, 2007).

Level 1: logit (Dream quality_ti_) = π_0i_ + *e*_ti_
Level 2: π_0i_ = β_00_ + β_01_ respective NDQ item.cgm_i_ + *r*_0i_

For the person-level analyses, we aggregated the dataset on the person level. For continuous level 1 variables, this resulted in means and mean squared successive differences (MSSD; Ebner-Priemer et al., 2009). For dichotomous level 1 data, frequencies were calculated. MSSDs have the advantage of reflecting the deviation of values across time more accurately than classical standard deviations, because they consider the time sequence. Instead of just using the deviation from the mean, the deviation of a certain value from the preceding value in the time sequence is calculated, incorporating information from the time sequence format.

For the multi-level (person and occasion) analyses, we calculated a GLMM for dichotomous data in line with the item-based analyses. The level 1 predictors were current well-being, alcohol consumption the day before, life event the day before, and the feeling of fullness before going to sleep. The three subscales of the NDQ were entered as level 2 predictors into the model.

Prior to the analyses we person-mean centered all level 1 variables and grand-mean centered all level 2 variables (Enders and Tofighi, 2007). Following the recommended procedure by Curran and Bauer (2011), we reintroduced the person-mean from level 1 centering at level 2. The person-mean centered variable then represents fluctuating state aspects whereas the person-mean itself represents stable trait aspects (cwc = values centered within context, i.e., around each participant’s mean; pm = person mean; cgm = centered grand-mean). For the final analysis, we used the following model for each of the three dummy coded dream qualities:

Level 1: logit (Dream quality_ti_) = π_0i_ + π_1i_ Well-being.cwc_ti_ + *e*_ti_

Level 2: π_0i_ = β_00_ + β_01_ Well-being.pm_i_ + β_02_ NDQ General.cgm_i_ + β_03_ NDQ Sleep.cgm_i_ + β_04_ NDQ Daytime Reality.cgm_i_ + *r*_0i_

Level 2: π_1i_ = β_10_ + *r*_1i_

## Results

To judge data quality, we asked for participants’ sex and age at the beginning of the ESM part of the study (assessed via the smartphone app) as well as at the end of the study, 22 days later in the final online questionnaire. Participants’ sex corresponded to 100% and age to 99%. Only one participant diverged with a difference of 7 years. Because the rest of the data from this participant was not suspect, we retained this participant in the data set.

The NDQ had satisfactory reliability (Cronbach α = 0.89). The subscales suggested by Böckermann et al. (2014) also elicited good to acceptable reliability scores: General distress (NDQ General; 5 items): α = 0.86; Impact on sleep (NDQ Sleep; 3 items): α = 0.66; Impact on daytime reality perception (NDQ Daytime Reality; 4 items): α = 0.73. In contrast to Böckermann et al. (2014), the impact on daytime reality perception subscale had in our case acceptable reliability (Böckermann et al., 2014; α = 0.51).

Although our data potentially allowed to differentiate between whether the nightmare was post-traumatic (i.e., due to a life-event) or idiopathic (no known cause), the number of nightmares after a life event was just *n* = 15. Therefore, we did not separate between those two types due to power reasons.

In general, only two participants could not remember any dream at all. All the other participants could remember up to every dream during the 22-day time frame (for dream frequency, see Table 1). On average, 10.2 dreams were recalled (*SD* = 5.3). The prevalence rate of recurrent nightmares (more than once a week) was 5% in our sample, which is very much in line with past research (e.g., Schredl, 2010). Furthermore, the correlation between nightmare distress and nightmare frequency was small to moderate (*r*s = 0.13–0.27, see Table 2) again in line with past research (e.g., Belicki, 1992). Participants who had at least one nightmare during the study phase (*n* = 48) reported an average of 2.1 nightmares within the 22 days (*SD* = 1.16, range 1–6). In general, we found no sex-specific effects regarding dream frequency [nightmares: *t*(89) = 1.09, *p* = 0.278, *d* = 0.25; nice dream: *t*(89) = -1.19, *p* = 0.239, *d* = -0.28; neutral dream: *t*(89) = 0.97, *p* = 0.337, *d* = -0.22] except for bad dreams. Women had a higher frequency of bad dreams compared to men, 2.3 vs. 1.3, respectively [*t*(89) = 2.07, *p* = 0.042, *d* = 0.48]. Furthermore, we found no age-specific effects regarding the frequency of the different dream qualities (all *r*s > -0.132, all *p*s > 0.213).

**Table 2:** Results of the person-level analyses (Spearman correlations).

	1.	2.	3.	4.	5.	6.	7.	8.	9.	10.	11.
1. NDQ general											
2. NDQ sleep	0.64***										
3. NDQ daytime reality	0.61***	0.41***									
4. Nightmare frequency	0.26*	0.27**	0.13								
5. Nice dream frequency	-0.15	-0.05	0.13	0.09							
6. Bad dream frequency	0.33**	0.26*	0.17	0.20^†^	-0.17						
7. Neutral dream frequency	0.06	0.01	-0.01	0.11	0.01	0.25*					
8. Mean well-being	-0.30**	-0.36***	-0.13	-0.09	0.33**	-0.27**	-0.10				
9. MSSD well-being	0.12	0.19^†^	0.15	0.17	0.19^†^	0.27**	-0.04	-0.15			
10. Life event frequency	0.14	0.20^†^	0.28**	0.01	0.16	0.19^†^	-0.06	-0.16	0.26*		
11. Alcohol frequency	0.06	0.03	0.03	0.04	0.10	-0.09	0.08	0.17	0.07	-0.12	
12. Food intake frequency	0.05	0.20^†^	0.29**	-0.06	0.16	0.04	-0.01	-0.06	0.27**	0.13	0.09

N = 92, ***p < 0.001, **p < 0.01, ^∗^p < 0.05, ^^†^^p < 0.10. NDQ, Nightmare Distress Questionnaire; MSSD, Mean Squared Successive Differences.

### Item-Level Analyses

To analyze if the items of the NDQ were associated with the occurrence of the different dream qualities (nice, bad, nightmare), we calculated GLMMs for each NDQ item. If an NDQ items measures distress regarding nightmares, then it should show a positive association with nightmares, no association with bad dreams, and a negative (or null) association with nice dreams. As can be seen from Table 3, none of the NDQ items only showed associations with nightmare dreams without showing an association with bad dreams as well.

**Table 3:** Results of the item-based analyses.

	Estimate of the fixed effect coefficient β_01_		
	Nice dream	Bad dream	Nightmare
**NDQ General distress subscale (NDQ General)**			
Item 5	-0.27	0.56***	0.49***
Item 6	-0.27	0.37**	0.43**
Item 7	-0.20	0.48***	0.34*
Item 8	-0.18	0.40**	0.46**
Item 13	-0.21	0.29*	0.30^†^
**NDQ Impact on sleep subscale (NDQ Sleep)**			
Item 1	0.02	0.31*	0.49**
Item 3	-0.26	0.49**	0.46*
Item 4	-0.08	0.24^†^	0.23
**NDQ Impact on daytime reality perception**			
**subscale (NDQ Daytime reality)**			
Item 2	0.08	0.32*	0.23
Item 9	0.15	0.25*	0.33*
Item 10	0.15	0.09	0.07
Item 11	0.25	0.12	0.21
**Excluded by** Böckermann et al., 2014			
Item 12	-0.41	0.28^†^	0.35^†^

*^∗∗∗^p < 0.001, ^∗∗^p < 0.01, ^∗^p < 0.05, ^^†^^p < 0.10. NDQ, Nightmare Distress Questionnaire.*

Furthermore, only one of the items (#9) of the NDQ Daytime Reality subscale showed an association with the occurrence of nightmares. All other items of the this subscale failed to show a significant association with nightmares. Counterintuitively, all Daytime Reality subscale items showed *positive* associations with nice dreams, though none was significant. This inconclusive pattern regarding the NDQ subscale Daytime Reality is in line with Böckermann et al. (2014) who stated that this subscale has probably little to do with the occurrence of nightmares.

All items of the NDQ General subscale showed a consistent association with nightmare dreams (see Table 3). However, the correlation for item #13 only approached significance, *p* < 0.10. This is also in line with Martínez et al. (2005) and Böckermann et al. (2014) who found that item 13 was problematic, in their case because of poor communalities. All items of the subscale did also show a consistent pattern of positive associations with bad dreams and negative associations with nice dream occurrence.

For the NDQ Sleep subscale, Item #1 and #2 showed associations with nightmare, but Item #4 actually failed. Furthermore, in line with Böckermann et al. (2014), we found that Item 12 was suspicious because it failed to reveal any association with different dream qualities (see Table 3).

### Person-Level Analyses

Next, we were interested if the NDQ is associated with the frequency of each dream quality as well as other suggested influences on dream quality (e.g., food intake before sleep, alcohol consumption, life events; for descriptives, see Table 1). If the NDQ has construct validity, then it should correlate with nightmares and bad dream frequency (positive correlations) as well as mean well-being (negative correlations; Blagrove et al., 2004). Furthermore, we should also find a higher fluctuation of well-being scores (represented by MSSD) due to nightmare distress. Regarding potential daytime influences, nightmare distress should be associated with high food intake before sleep, alcohol consumption, and the occurrence of life events. Intercorrelations of these variables are shown in Table 2.

NDQ subscales showed significant and substantial inter-correlations (see Böckermann et al., 2014). The frequency of different dream qualities was unrelated except for a positive correlation between nightmare and bad dream frequency that almost reached statistical significance (*r* = 0.20, *p* < 0.10). Interestingly, frequently having nice dreams does not lower the probability of having a bad dream or nightmare. This supports the assumption that dreams are independent from each other with regard to their quality.

Nightmare frequency was unrelated to trait- and state-levels of well-being, which was surprising. For nice and bad dream frequency, we found significant correlations in the expected directions (nice dreams were *positively* associated with trait well-being, bad dreams *negatively* associated with trait well-being).

Regarding construct validity NDQ General and NDQ Sleep showed convergent as well as discriminant validity by being positively correlated with nightmare and bad dream frequency, and negatively with the mean well-being during the 3-week time frame of data collection. NDQ General and NDQ Sleep were not significantly correlated with nice dream frequency or fluctuation of well-being over time. Descriptively, they did show the expected associations, though. NDQ Daytime Reality failed to show any significant correlations.

To sum up, we found construct validity for NDQ General and NDQ Sleep but not for NDQ Daytime Reality. Furthermore, we found that the NDQ was unable to differentiate between nightmare and bad dream frequency.

### Multi-Level (Person and Occasion) Analyses

In a further step, we wanted to know if there is an association of the three subscales of the NDQ with the probability of having a certain type of dream. First, alcohol consumption, occurrence of a life event, and feelings of fullness did not show any significant effects on dream quality in any of the analyses (except for a counterintuitive small effect of alcohol consumption on the probability of not having a bad dream) and were therefore discarded to keep the models parsimonious.

As can be seen in Table 4, a nice dream was associated with higher well-being the next morning whereas a bad dream and nightmare was associated with significantly lower well-being. Regarding the NDQ, only the NDQ General subscale had any consistent predictive value for dream quality. Higher general nightmare distress was associated with a lower chance for a nice dream, but a higher chance for a bad dream and nightmare (although not significant for a nightmare). NDQ Sleep had no predictive value for any type of dreams and NDQ Daytime Reality did show a reversed, counterintuitive value for dream quality, i.e., the higher NDQ Daytime Reality the *higher* was the chance of having a nice dream.

**Table 4:** Results of the multi-level analyses.

Outcome Predictor	Fixed				Random	
	Coef.	Est.	*SE*	*z*	Coef.	*SD*
**Nice dream**				
Intercept	β_00_	-4.38			*r*_0_*_i_*	1.11
Well-being.cwc	β_10_	0.02	<0.01	3.29***	*r*_1_*_i_*	0.02
Well-being.pm	β_01_	0.04	0.01	2.37*		
NDQ general	β_02_	-0.94	0.37	-2.57*		
NDQ sleep	β_03_	0.39	0.29	1.35		
NDQ daytime reality	β_04_	0.70	0.29	2.41*		
**Bad dream**				
Intercept	β_00_	-1.35			*r*_0_*_i_*	0.76
Well-being.cwc	β_10_	-0.02	<0.01	-2.58**	*r*_1_*_i_*	0.03
Well-being.pm	β_01_	-0.01	0.01	-1.26		
NDQ general	β_02_	0.68	0.23	3.00**		
NDQ sleep	β_03_	-0.07	0.23	-0.29		
NDQ daytime reality	β_04_	-0.07	0.23	-0.33		
**Nightmare**				
Intercept	β_00_	-3.14			*r*_0_*_i_*	1.05
Well-being.cwc	β_10_	-0.04	0.01	-4.40***	*r*_1_*_i_*	0.02
Well-being.pm	β_01_	>-0.01	0.01	-0.01		
NDQ general	β_02_	0.51	0.29	1.75^†^		
NDQ sleep	β_03_	0.30	0.28	1.05		
NDQ daytime reality	β_04_	-0.04	0.28	-0.15		

Coef., Coefficient from multilevel Equations; Est., Estimate; Well-being.cwc, person-mean centered well-being; Well-being.pm, person mean of well-being reintroduced into the model as level 2 variable. NDQ subscales (level 2) were grand-mean centered. NDQ, Nightmare Distress Questionnaire. ***p < 0.001, **p < 0.01, ^∗^p < 0.05, ^^†^^p < 0.10.

## Discussion

In the present methodological study, we analyzed the validity of the NDQ, being one of the most used measures of nightmare distress. To achieve this, we implemented data from an experience sampling design. We assessed dream quality and further related variables over time, investigating the contextual associations as well as their associations with the NDQ. The results can be summarized as follows:

The items from the NDQ General subscale were able to differentiate between dream qualities (negative vs. positive) slightly better (except Item 13) than the items from the NDQ Sleep subscale. Similar to the NDQ Sleep subscale, it showed significant correlations with nightmare and bad dream frequencies, and convergent validity with well-being (only the mean, not the fluctuations over time). Compared to the other NDQ subscales, NDQ General was the best predictor of the different dream qualities in the multi-level view.

The items from the NDQ Sleep subscale were also capable of differentiating between positive and negative dream qualities (except Item #4). The subscale showed significant correlations with nightmare and bad dream frequencies, and convergent validity with well-being (again only the mean, not the fluctuations over time). The subscale was not capable of predicting any kind of dream quality in the multi-level analyses.

Finally, the NDQ Daytime Reality subscale does not seem to be associated with nightmare distress at all. First, the items belonging to that subscale were not clearly capable of differentiating between negative and positive dream qualities in general (except Item #9, but also revealed a counterintuitive positive association with nice dream occurrence). Although this subscale showed substantial correlations with the other two NDQ subscales (NDQ General, NDQ Sleep), it failed to show any significant associations with dream frequencies. Interestingly, this subscale showed significant correlations with the frequency of life events and feeling of fullness frequency, in contrast to the other NDQ subscales.

Nevertheless, because the NDQ General as well as the NDQ Sleep subscale did not show any substantial associations with these variables, it remains unclear if this can be interpreted as a sign of convergent validity for the NDQ Daytime Reality subscale. Furthermore, from the multi-level view, NDQ Daytime Reality had a *positive* effect on the probability of having a nice dream, not, as would have been expected, *negative* dreams (bad dream, nightmare). Although further research is needed here, it seems that NDQ Daytime Reality is probably not an integral concept of nightmare distress (see also Böckermann et al., 2014). If at all, NDQ Daytime Reality might reflect a concept which is indirectly associated with nightmare distress.

To sum up, item-based analyses revealed that the NDQ did not really differentiate between a bad dream and a nightmare. This is supported by the multi-level analyses where the NDQ had similar predictive value for the bad dream and nightmare (descriptively, even higher for the bad dream). Therefore, the NDQ might be rather a measure of *negative* dream distress including bad dreams that are not nightmares.

Furthermore, our analyses suggest perhaps dropping Item 13 from the NDQ General subscale, Item 4 from the NDQ Sleep subscale, as well as dropping the whole NDQ Daytime Reality subscale. In our study, we only found few associations with dream quality and other indicators for this subscale, casting doubt on its validity and usefulness.

### Predictors of Negative Dreams

Although past research found associations of negative dreams with well-being (e.g., Levin and Fireman, 2002), occurrence of life events (e.g., Dunn and Barrett, 1988), alcohol consumption (e.g., Munezawa et al., 2011), and food intake (e.g., Nielsen and Powell, 2015), we only found some significant associations with well-being (see Table 2). Participants with a higher frequency of nice dreams and lower frequency of bad dreams had higher well-being on average. Bad dream frequency was associated with a higher fluctuation of well-being over time. All the other potential predictors failed to show significant effects. Besides the possibility that indeed H_0_ is true, there might be other explanations for these findings. First, alcohol consumption was only assessed in the 3-week time frame, i.e., it might be not representative for the time outside this time frame. Furthermore, the alcohol intake of the night before was assessed the next day. This measure has been shown to be less accurate than real-time assessment (Monk et al., 2015). Second, the definition of life events was very broad, beginning with minor conflicts with the partner to severe life events such as the death of a beloved person. Focusing on severe life events might have shown effects. Third, the effect of food intake onto dreams is in general a rather weak finding mostly of anecdotal origin (Nielsen and Powell, 2015). In our study, we did not find any effects for feelings of fullness, except for NDQ Daytime reality, but its validity is unclear.

### Limitations

Although we collected over 3,500 data points from 92 participants, 44 retests, and a mean compliance rate of 90.3%, the design was slightly underpowered to detect small to medium effects (ICC = 0.3, α = 5%, power = 80%, conservative power calculation based on the recommendation by Twisk (2006), (p. 123ff), 80% power reached for correlations larger than 0.16). Nevertheless, convergent validity requires substantial correlations. Therefore, the low power for small effects only reduces the exploratory power of divergent validity where weak to null correlations are expected.

Furthermore, our results are limited by the fact that our sample was relatively young and consists mainly of women. Moreover, we had a non-clinical sample. In practice, the NDQ might be mostly used to screen for distress in patients with nightmares (nightmare diagnoses ICD-10: F51.5). Associations between the NDQ and the prospectively assessed items might be different in actual patients.

### Future Directions

It is interesting that the frequencies of the different dream qualities were almost unrelated. This supports the assumption that a certain dream type on a particular day does not influence the occurrence of a certain dream type in the following night (such as dream-lag effects; e.g., Henley-Einion and Blagrove, 2014), i.e., they seem to be isolated events with minimal “spillover” effects, if any. Future research might address this in more detail.

Furthermore, in line with Böckermann et al. (2014), we found that distress was produced not only by nightmares, but also by bad dreams (see item-based analyses in Table 3). The awakening, which distinguishes nightmares from bad dreams, did not elicit any differences in distress. This could be due to two reasons: First, the NDQ might really be a global measure of “negative sleep distress.” Second, participants who are asked about nightmare distress retrospectively might not be capable to differentiate between distresses elicited by nightmares as compared to bad dreams. Because the questions in the NDQ explicitly focus on the frequency of nightmare-related aspects (e.g., falling to sleep again, negative impact on well-being) and not bad dreams *per se*, we would rather think that the second reasoning is true, i.e., because of the retrospective remembering of nightmare events, participants are not capable to differentiate between bad dreams and nightmares anymore. Future research could investigate the differentiation further to try to discern distress from bad dreams and nightmares.

Because prevalence rates of nightmares in the population are comparatively high, future research could try to assess situation-dependent state-aspects of nightmare distress in the morning after a nightmare took place using an experience sampling design with an event-based sampling procedure. After several of these events, a mean of these NDQ state scale measurements can be calculated which might be a better predictor of nightmare-related aspects than the classical trait-based NDQ (for a similar discussion about the dimensional structure of state- and trait-aspects, see Schimmack et al., 2000).

## Author Contributions

SS and TK conceived and designed the study, acquired the data, and wrote the manuscript. SS analyzed and interpreted the data.

## Conflict of Interest Statement

The authors declare that the research was conducted in the absence of any commercial or financial relationships that could be construed as a potential conflict of interest.
